# Supplementary material for: A method to synthesize analytical rhodoquinone standards for quantitative analysis in tissue specimen
Source: bioRxiv. 2026 Jul 4:2026.05.04.722805. Originally published 2026 May 7. Preprint. [Version 2] doi: 10.64898/2026.05.04.722805 (PMC13174367; doi:10.64898/2026.05.04.722805)
Supplement: Supplement 1 — Figure S1. Proton (1H) NMR (500 MHz, CDCl3) spectra of isorhodoquinone-10. Figure S2. Carbon 13 (13C) NMR (126 MHz, CDCl3) spectra of isorhodoquinone-10. Figure S3. Distortionless Enhancement by Polarization Transfer (DEPT) NMR (126 MHz, CDCl3) spectra of isorhodoquinone-10. Figure S4. Heteronuclear Single Quantum Coherence (HSQC) NMR analysis of isorhodoquinone-10. Figure S5. Heteronuclear Multiple Bond Correlation (HMBC) NMR analysis of isorhodoquinone-10. Figure S6. Proton (1H) NMR (500 MHz, CDCl3) spectra of rhodoquinone-10. Figure S7. Carbon 13 (13C) NMR (126 MHz, CDCl3) spectra of rhodoquinone-10. Figure S8. Distortionless Enhancement by Polarization Transfer (DEPT) NMR (126 MHz, CDCl3) spectra of rhodoquinone-10. Figure S9. Heteronuclear Single Quantum Coherence (HSQC) NMR analysis of rhodoquinone-10. Figure S10. Heteronuclear Multiple Bond Correlation (HMBC) NMR analysis of rhodoquinone-10. Figure S11. Stability of rhodoquinone-10 standard under light and methanol exposure. (A) Relative rhodoquinone-10 levels in standard samples diluted in either 80:20 ethanol:hexane or 100% methanol with 2 mM ammonium formate and incubated for 1 hour under dark, ambient light, or ultraviolet (UV) light conditions. Integrated adducts includes [M+H]+: 848.6921 m/z, [M+Na]+: 870.6740 m/z, and [M+NH4]+: 865.7186 m/z. (B) Relative rhodoquinone-10 levels in standards diluted in either 80:20 ethanol:hexane or 100% methanol with 2 mM ammonium formate and incubated for 1 hour under dark, ambient light, or ultraviolet (UV) light conditions. Integrated adducts includes [M+2H]+: 851.7155 m/z, [M+H]+: 850.7077 m/z, and [M+Na]+: 872.6897 m/z. (C) Relative rhodoquinol-10 to rhodoquinone-10 ratios of standards diluted in either 80:20 ethanol:hexane or 100% methanol with 2 mM ammonium formate and incubated for 1 hour under dark, ambient light, or ultraviolet (UV) light conditions. (D) Relative ubiquinone-10 generated from resuspending standards in either 80:20 ethanol:hexane or 100% methano [file media-1.pdf]

Figure S1

^{1}H NMR ($CDCl_3$, 500 MHz)
Compound 1
isoRhodoquinone-10

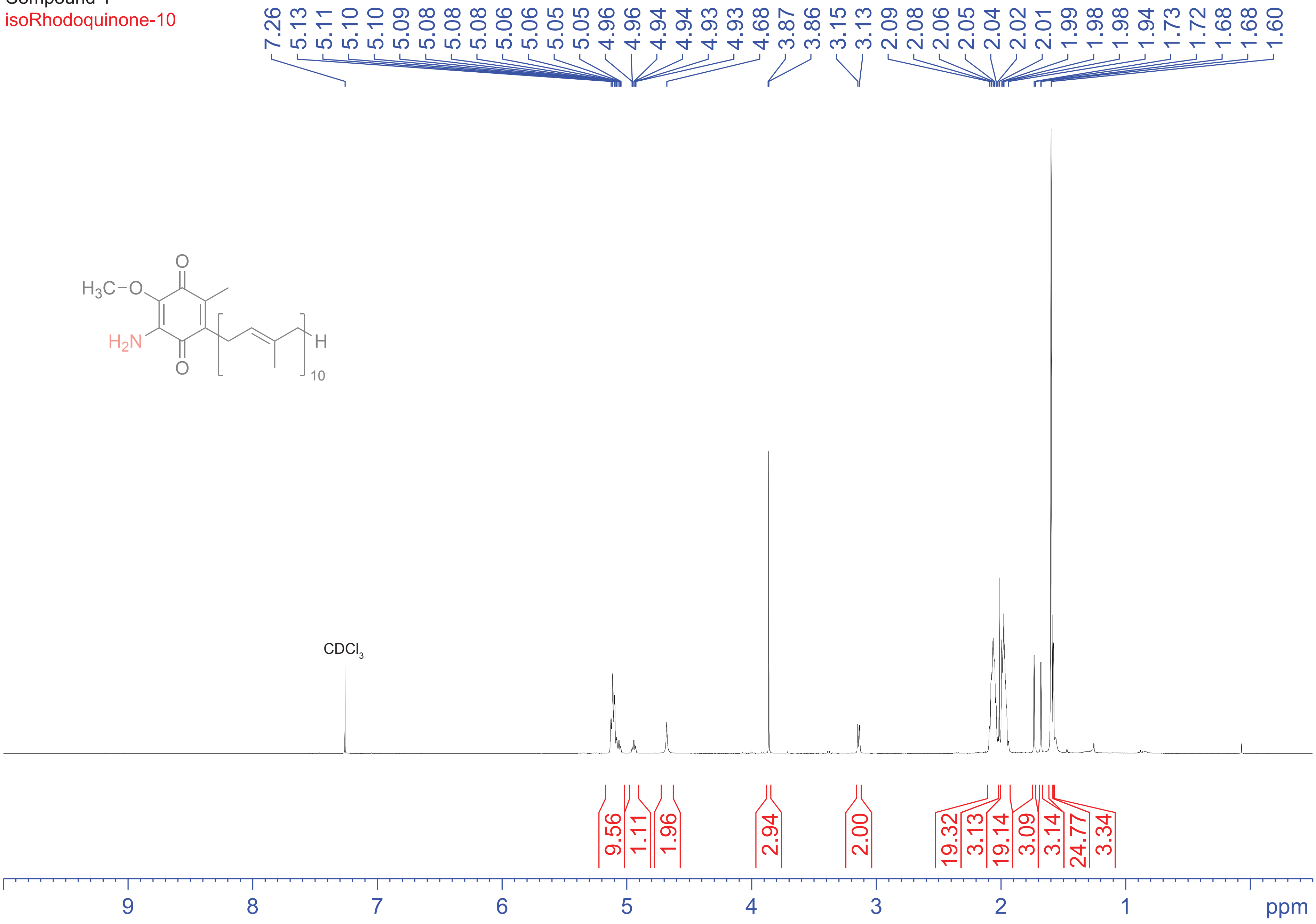

Figure S2

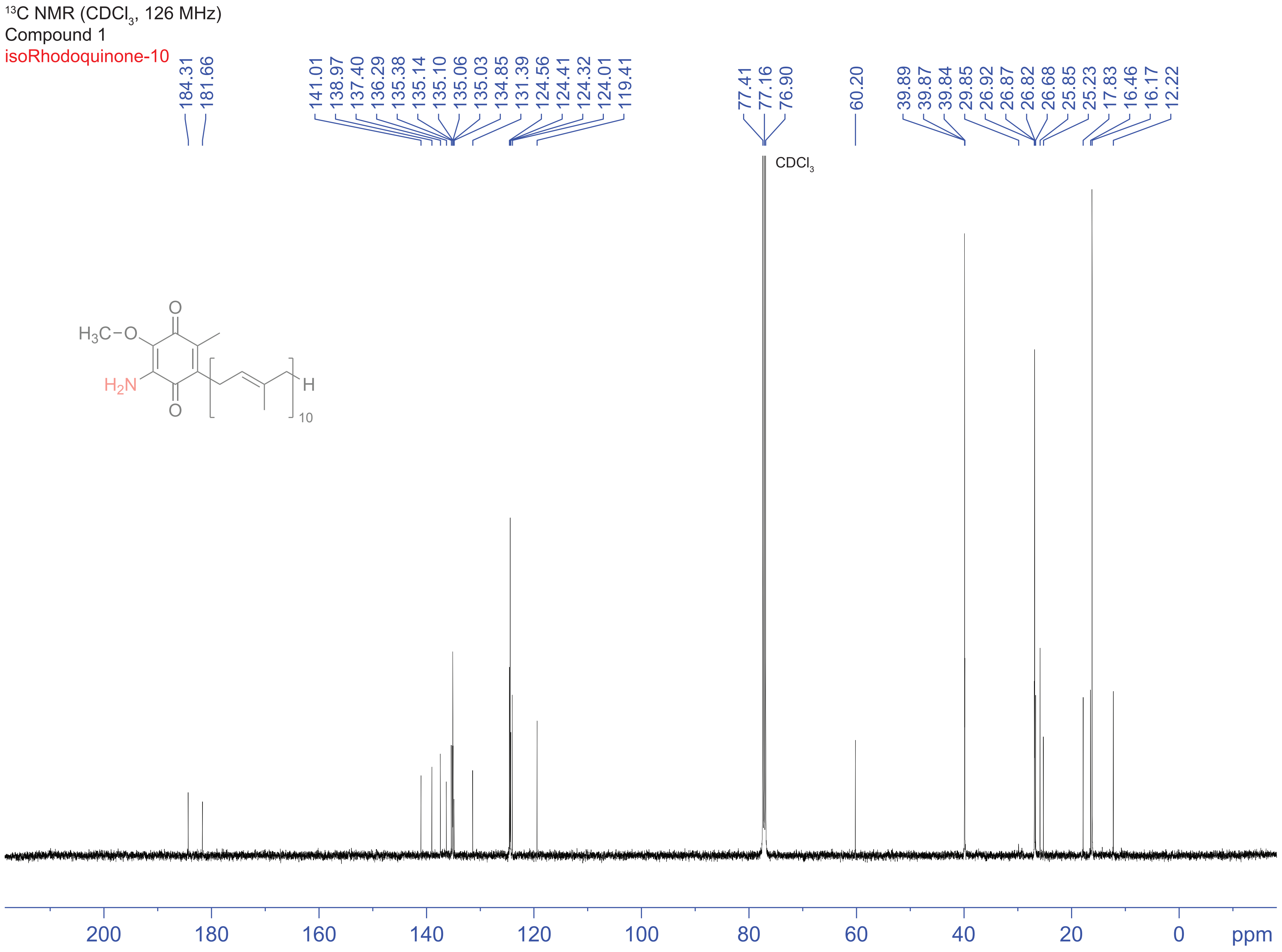

^{13}C NMR ($CDCl_3$, 126 MHz)
Compound 1
isoRhodoquinone-10
184.31
181.66
141.01
138.97
137.40
136.29
135.38
135.14
135.10
135.06
135.03
134.85
131.39
124.56
124.41
124.32
124.01
119.41
77.41
77.16
76.90
60.20
39.89
39.87
39.84
29.85
26.92
26.87
26.82
26.68
25.85
25.23
17.83
16.46
16.17
12.22
CDCl3
H3C−O
O
O
H2N
H
10
200
180
160
140
120
100
80
60
40
20
0
ppm

Figure S3

DEPT NMR ($CDCl_3$, 126 MHz)
Compound 1
isoRhodoquinone-10

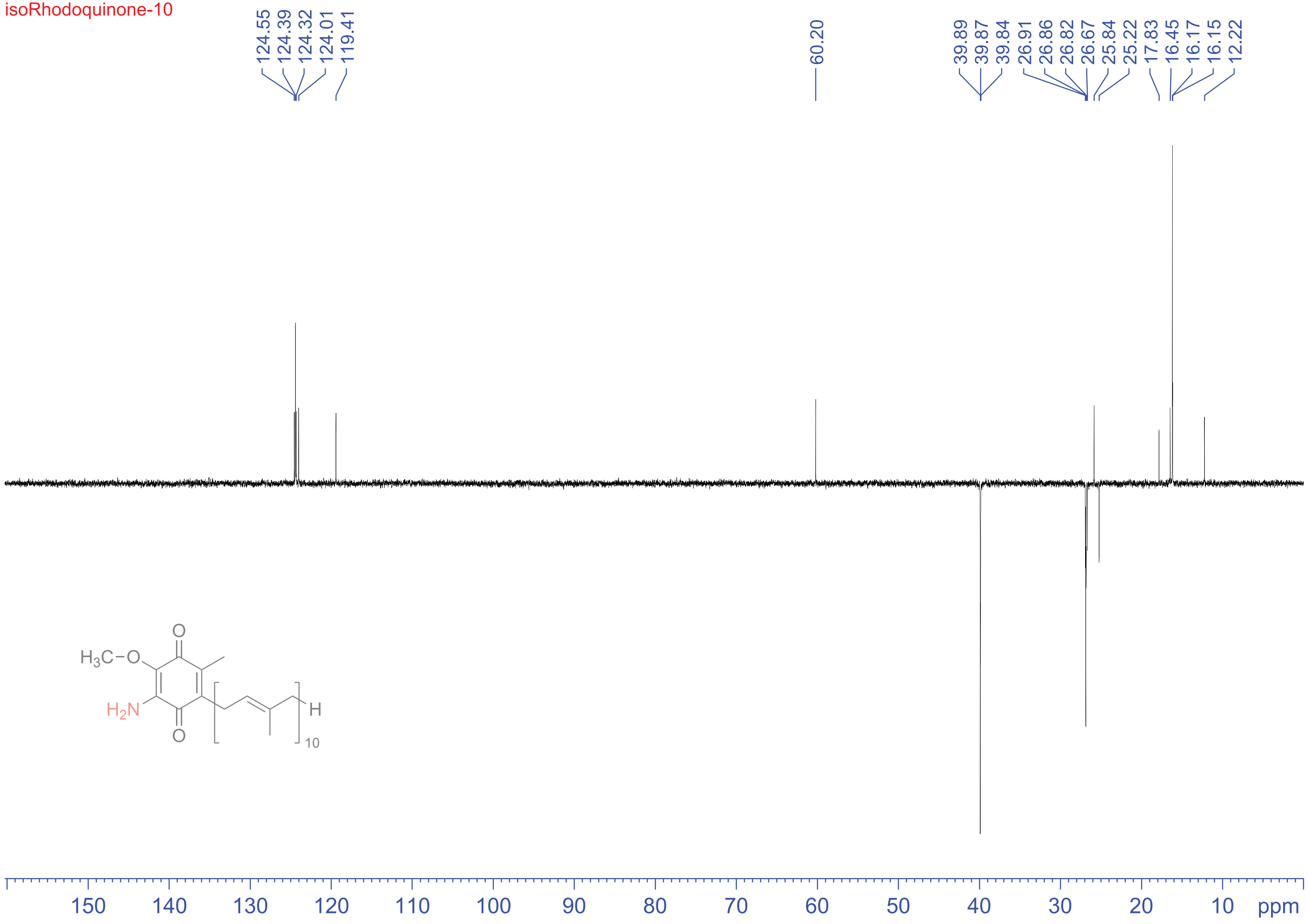

Figure S4

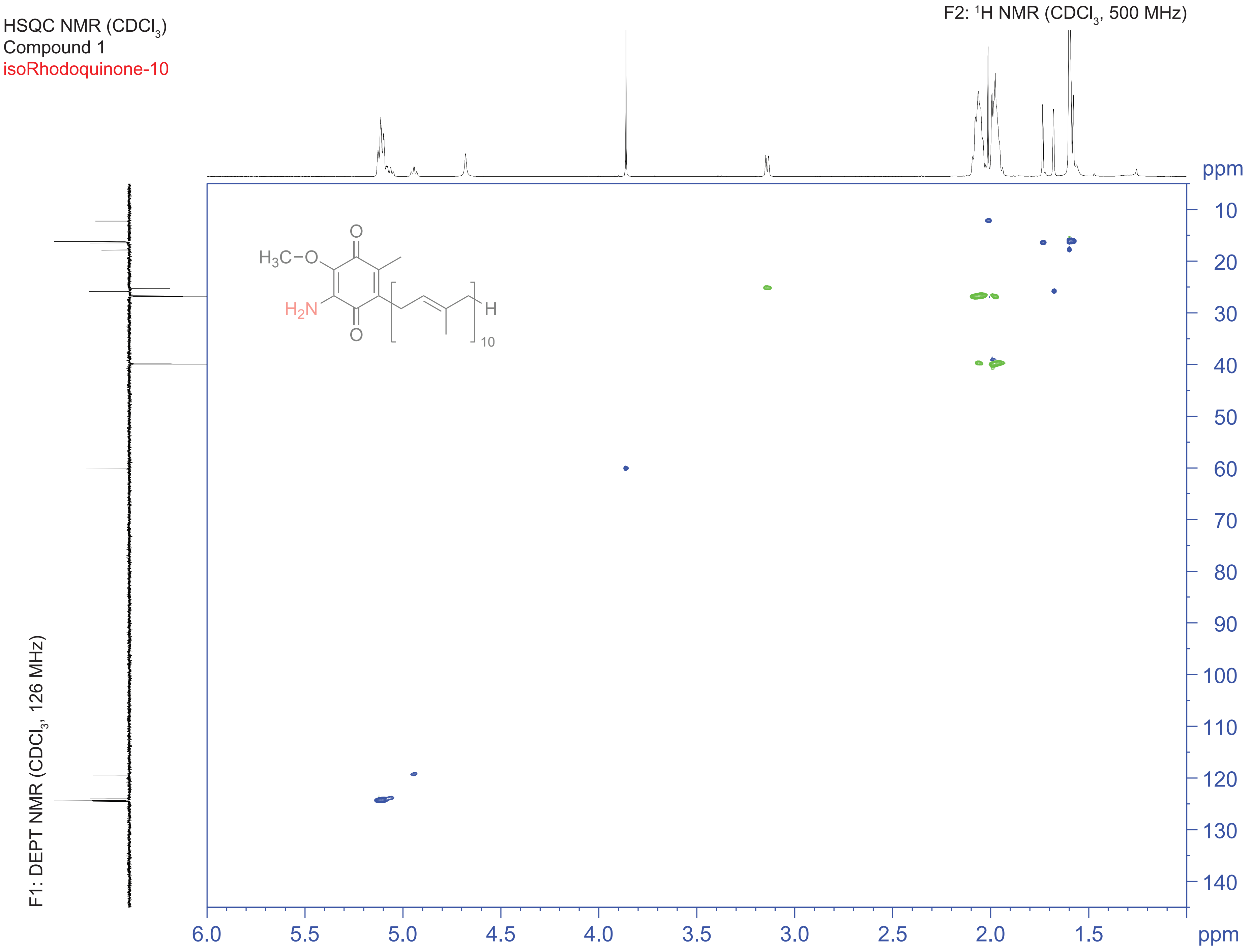
HSQC NMR (CDCl3)
Compound 1
isoRhodoquinone-10
F2: 1H NMR (CDCl3, 500 MHz)
F1: DEPT NMR (CDCl3, 126 MHz)
H3C−O
H2N
O
H
10
ppm
10
20
30
40
50
60
70
80
90
100
110
120
130
140
6.0
5.5
5.0
4.5
4.0
3.5
3.0
2.5
2.0
1.5

Figure S5

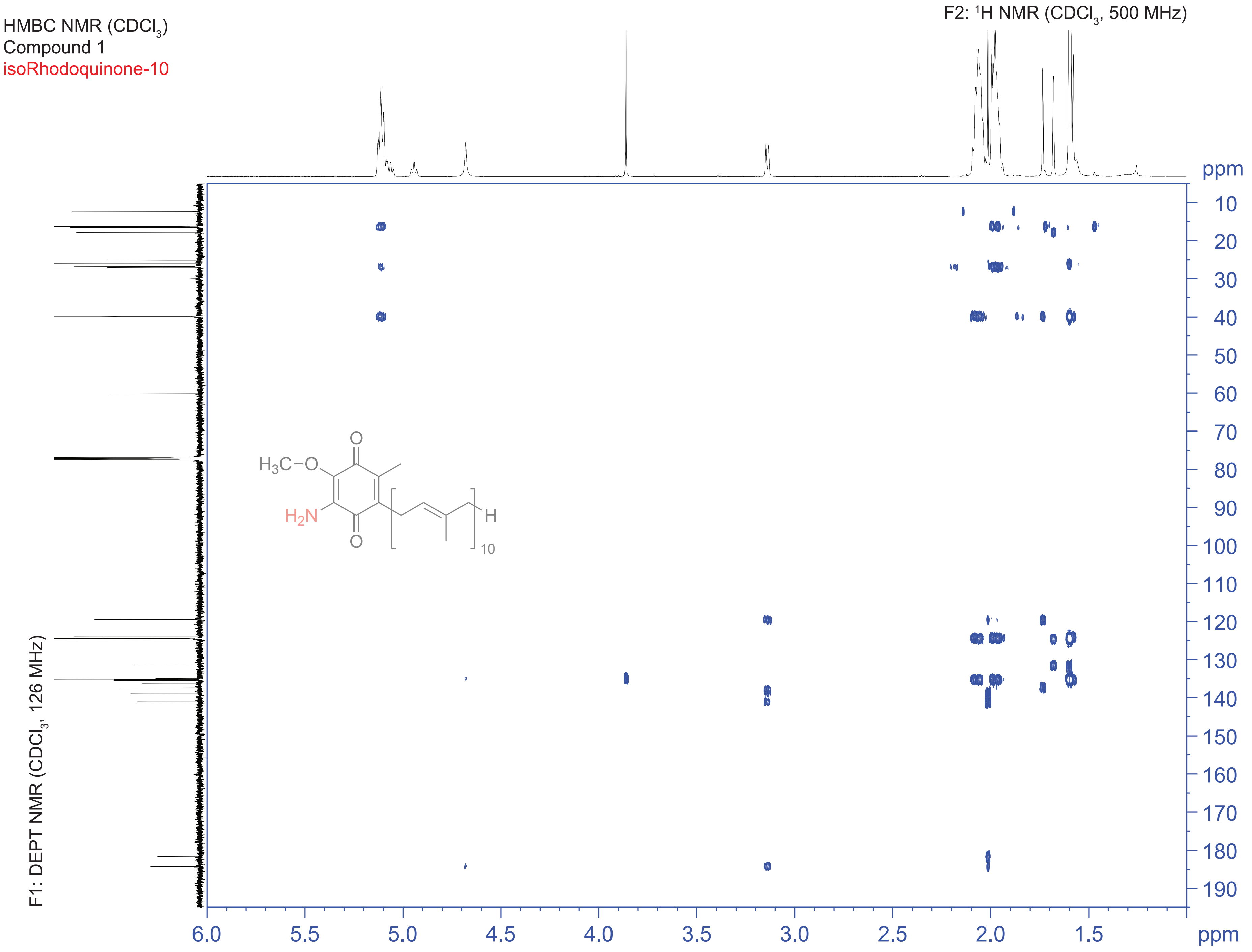
HMBC NMR (CDCl3)
Compound 1
isoRhodoquinone-10
F2: 1H NMR (CDCl3, 500 MHz)
F1: DEPT NMR (CDCl3, 126 MHz)
ppm
10
20
30
40
50
60
70
80
90
100
110
120
130
140
150
160
170
180
190
6.0
5.5
5.0
4.5
4.0
3.5
3.0
2.5
2.0
1.5
ppm
H3C−O
H2N
O
O
H
10

Figure S6

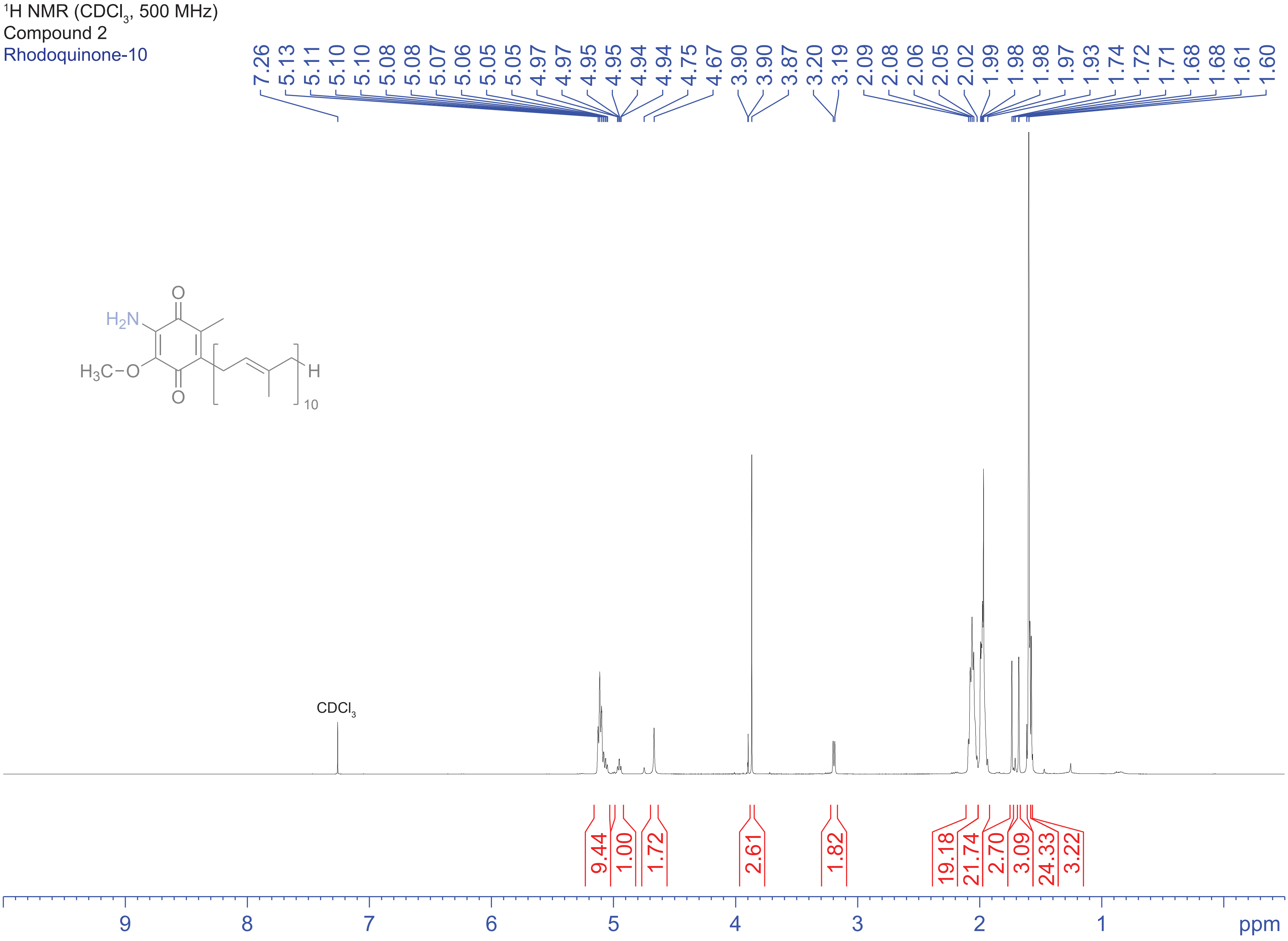

^{1}H NMR ($CDCl_3$, 500 MHz)
Compound 2
Rhodoquinone-10
7.26 5.13 5.11 5.10 5.10 5.08 5.08 5.07 5.06 5.05 5.05 4.97 4.97 4.95 4.95 4.94 4.94 4.75 4.67 3.90 3.90 3.87 3.20 3.19 2.09 2.08 2.06 2.05 2.02 1.99 1.98 1.98 1.97 1.93 1.74 1.72 1.71 1.68 1.68 1.61 1.60
H_2N
H_3C-O
O
O
H
10
$CDCl_3$
9.44 1.00 1.72 2.61 1.82 19.18 21.74 2.70 3.09 24.33 3.22
9 8 7 6 5 4 3 2 1 ppm

Figure S7

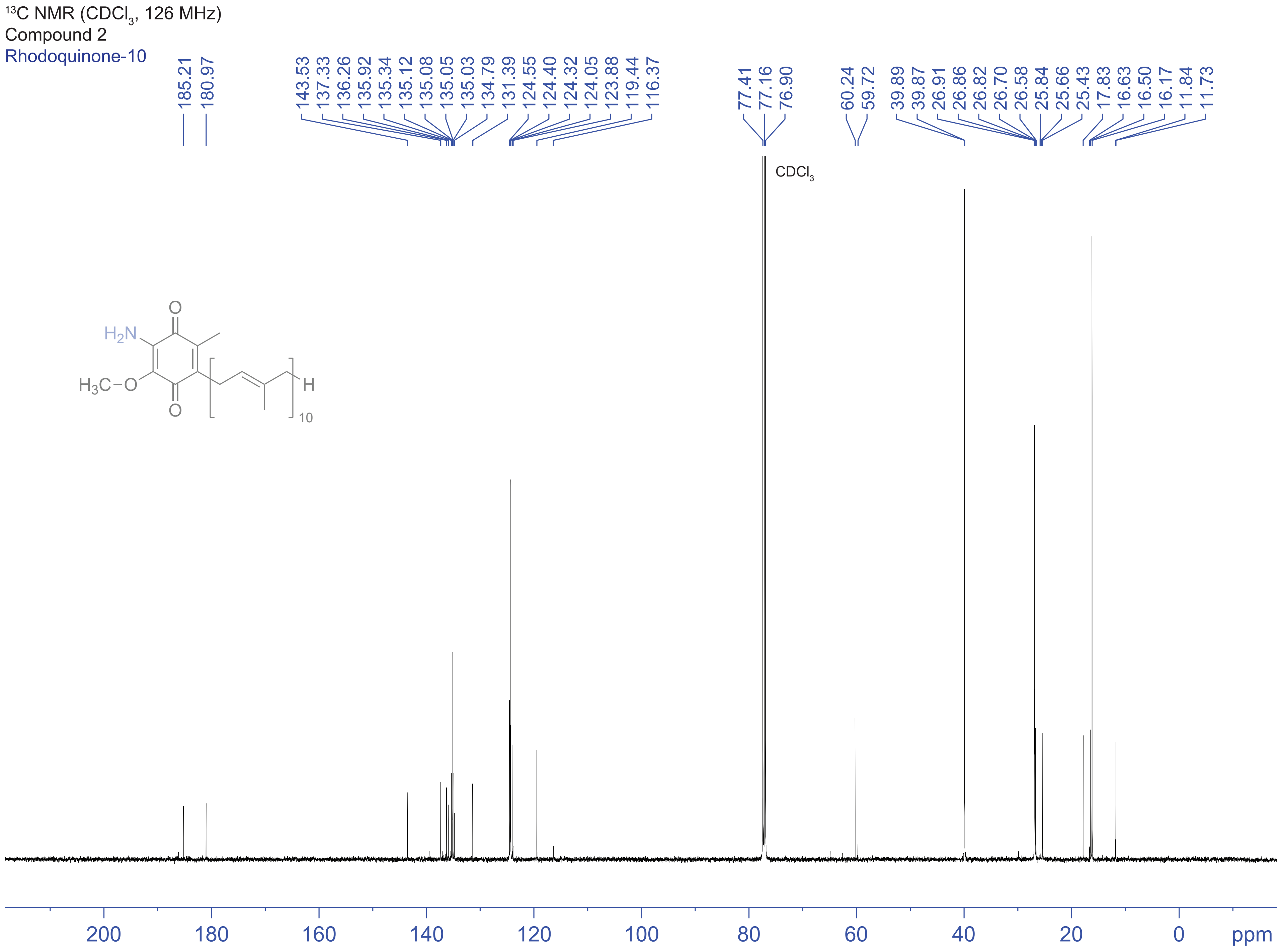

^{13}C NMR ($CDCl_3$, 126 MHz)
Compound 2
Rhodoquinone-10
185.21
180.97
143.53
137.33
136.26
135.92
135.34
135.12
135.08
135.05
135.03
134.79
131.39
124.55
124.40
124.32
124.05
123.88
119.44
116.37
77.41
77.16
76.90
60.24
59.72
39.89
39.87
26.91
26.86
26.82
26.70
26.58
25.84
25.66
25.43
17.83
16.63
16.50
16.17
11.84
11.73
CDCl3
H2N
H3C−O
O
O
H
10
200
180
160
140
120
100
80
60
40
20
0
ppm

Figure S8

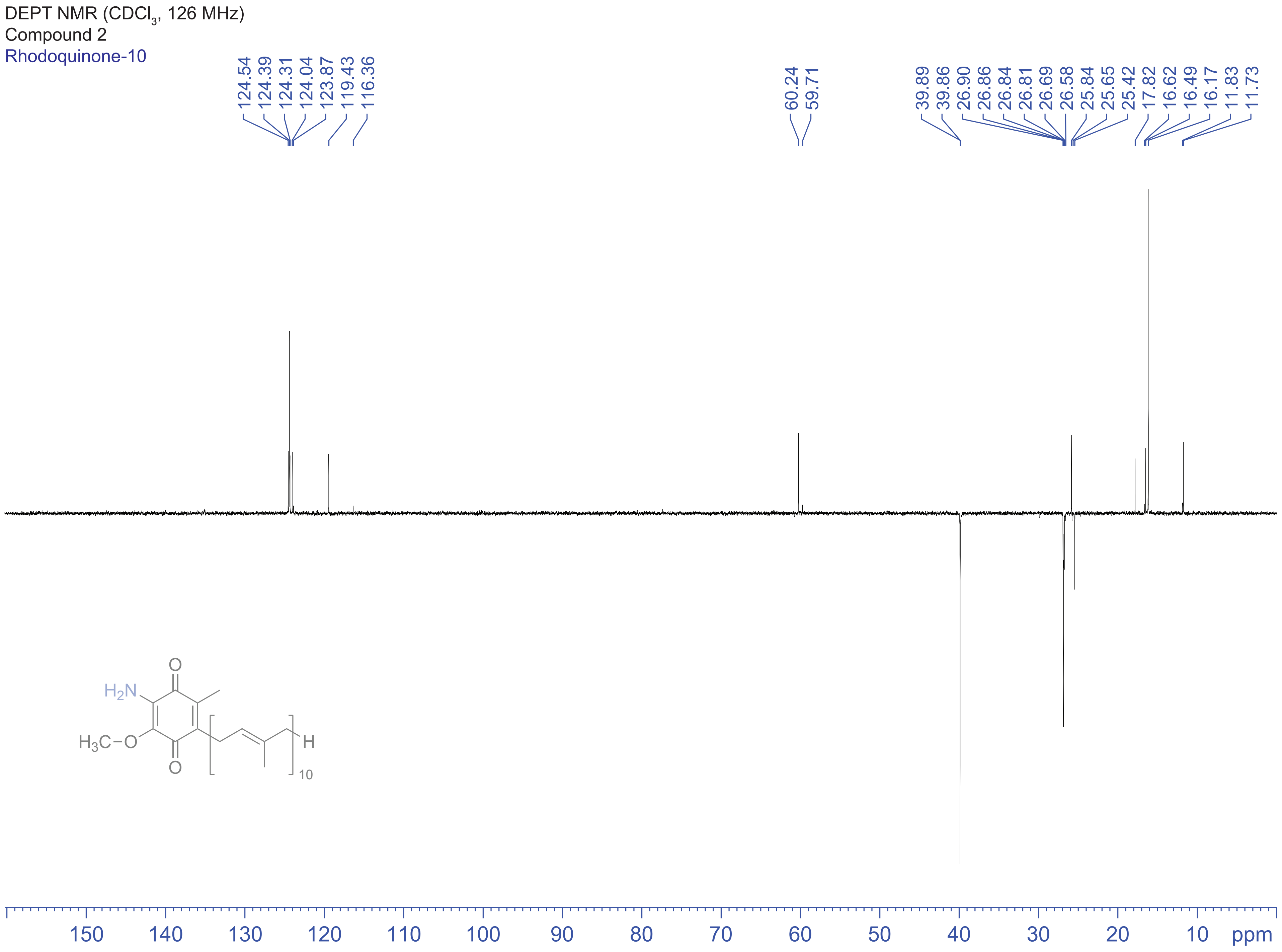

DEPT NMR ($CDCl_3$, 126 MHz)
Compound 2
Rhodoquinone-10
124.54
124.39
124.31
124.04
123.87
119.43
116.36
60.24
59.71
39.89
39.86
26.90
26.86
26.84
26.81
26.69
26.58
25.84
25.65
25.42
17.82
16.62
16.49
16.17
11.83
11.73
150
140
130
120
110
100
90
80
70
60
50
40
30
20
10
ppm
O
H2N
H3C−O
O
H
10

Figure S9

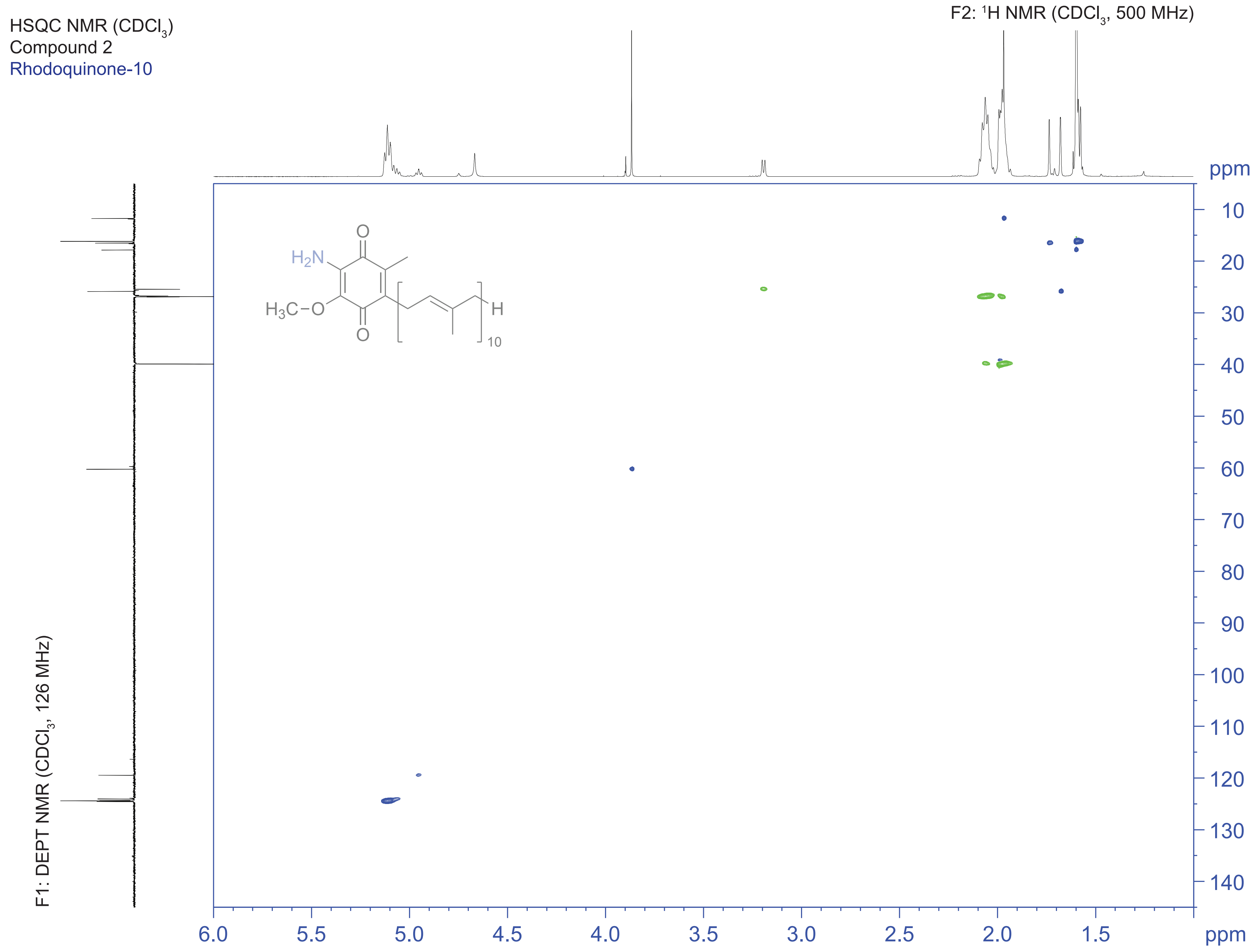
HSQC NMR ($CDCl_3$)
Compound 2
Rhodoquinone-10
F2: ^1H NMR ($CDCl_3$, 500 MHz)
F1: DEPT NMR ($CDCl_3$, 126 MHz)
ppm
10
20
30
40
50
60
70
80
90
100
110
120
130
140
6.0
5.5
5.0
4.5
4.0
3.5
3.0
2.5
2.0
1.5
ppm
O
H_2N
H_3C-O
O
H
10

Figure S10

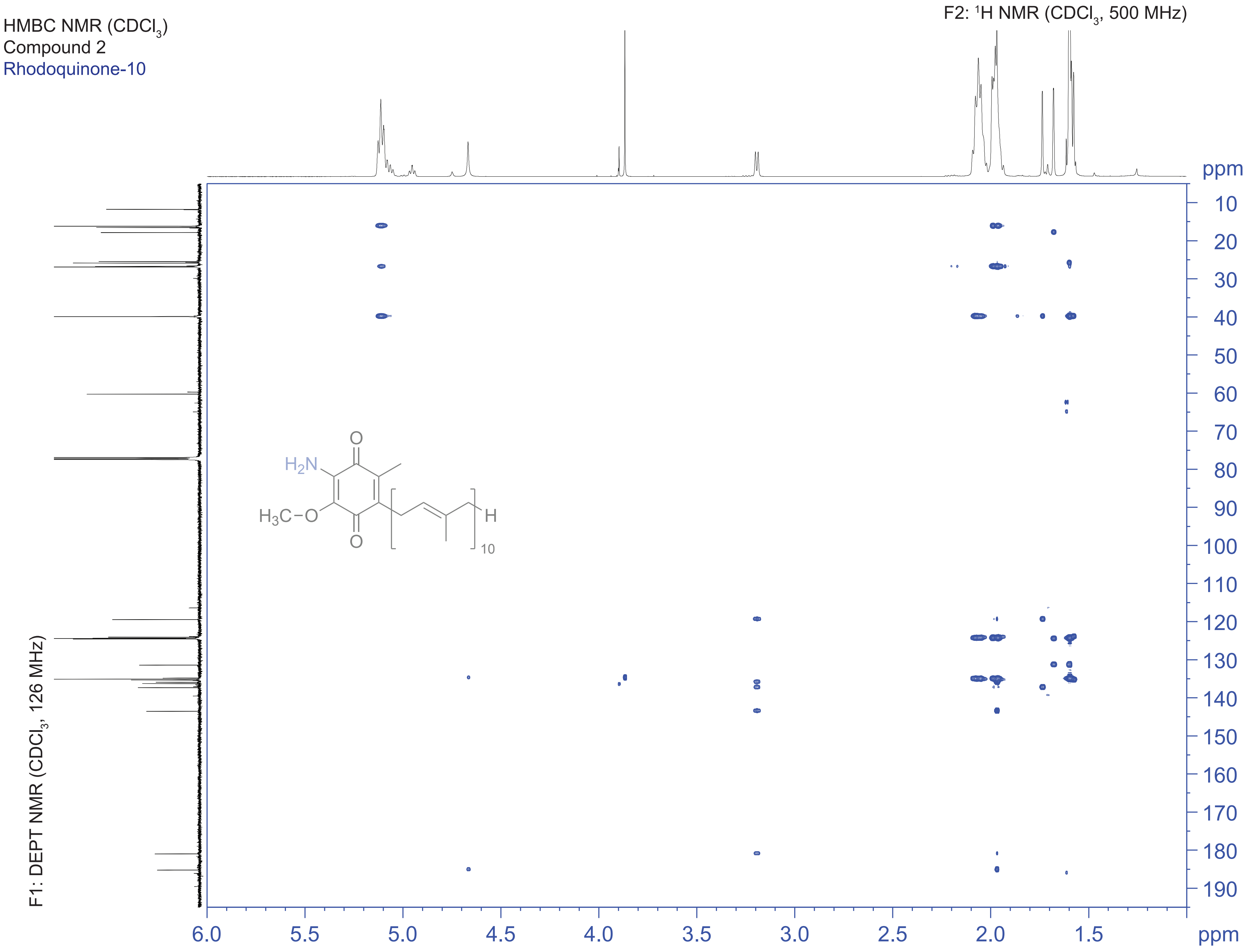
HMBC NMR (CDCl3)
Compound 2
Rhodoquinone-10
F2: 1H NMR (CDCl3, 500 MHz)
F1: DEPT NMR (CDCl3, 126 MHz)
ppm
10
20
30
40
50
60
70
80
90
100
110
120
130
140
150
160
170
180
190
6.0
5.5
5.0
4.5
4.0
3.5
3.0
2.5
2.0
1.5
ppm
H2N
H3C–O
O
O
H
10

Figure S11

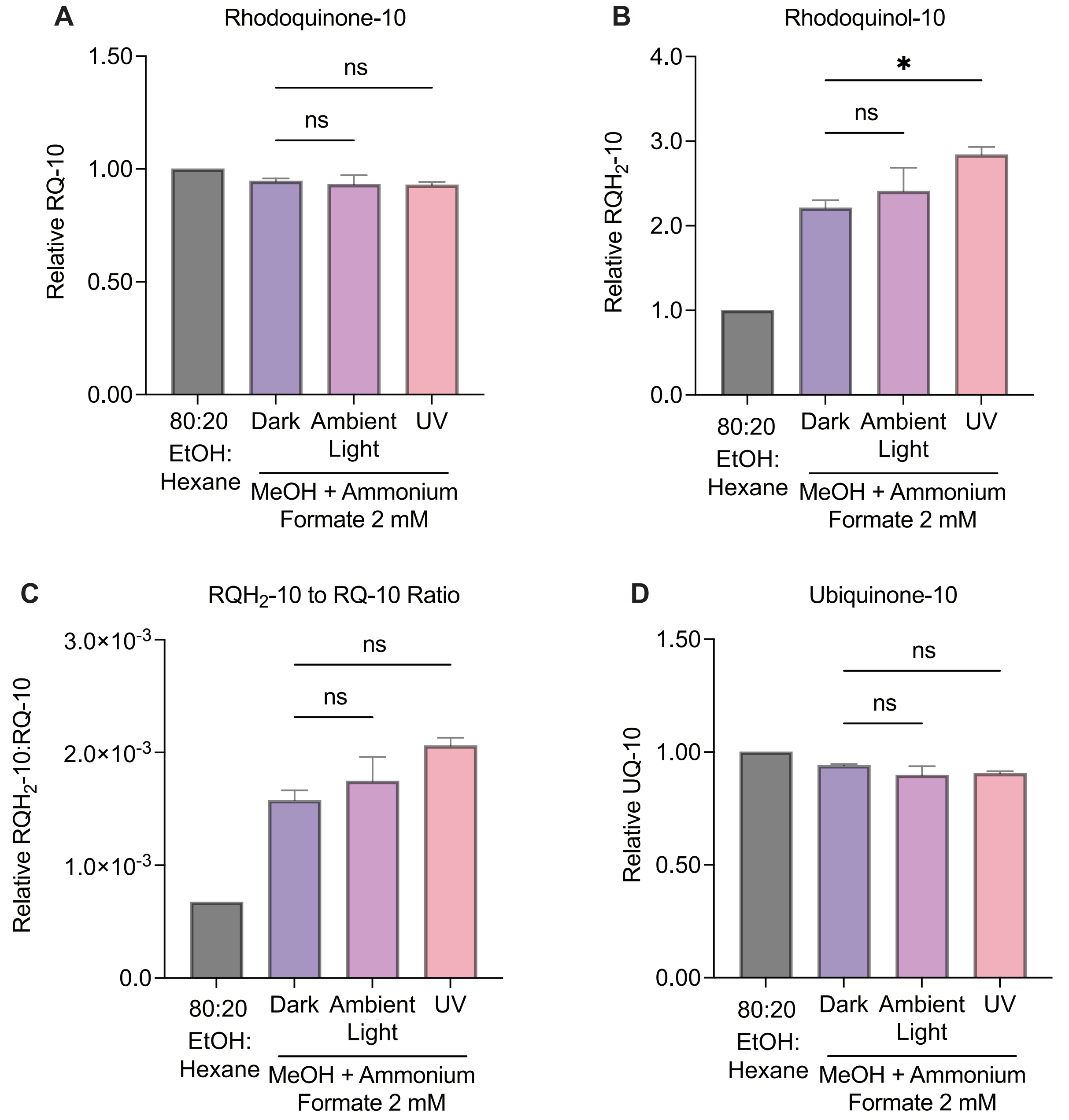
A
Rhodoquinone-10
Relative RQ-10
1.50
1.00
0.50
0.00
ns
ns
80:20 EtOH: Hexane
Dark
Ambient Light
UV
MeOH + Ammonium Formate 2 mM
B
Rhodoquinol-10
Relative RQH2-10
4.0
3.0
2.0
1.0
0.0
*
ns
80:20 EtOH: Hexane
Dark
Ambient Light
UV
MeOH + Ammonium Formate 2 mM
C
RQH2-10 to RQ-10 Ratio
Relative RQH2-10:RQ-10
3.0×10-3
2.0×10-3
1.0×10-3
0.0
ns
ns
80:20 EtOH: Hexane
Dark
Ambient Light
UV
MeOH + Ammonium Formate 2 mM
D
Ubiquinone-10
Relative UQ-10
1.50
1.00
0.50
0.00
ns
ns
80:20 EtOH: Hexane
Dark
Ambient Light
UV
MeOH + Ammonium Formate 2 mM

Figure S12

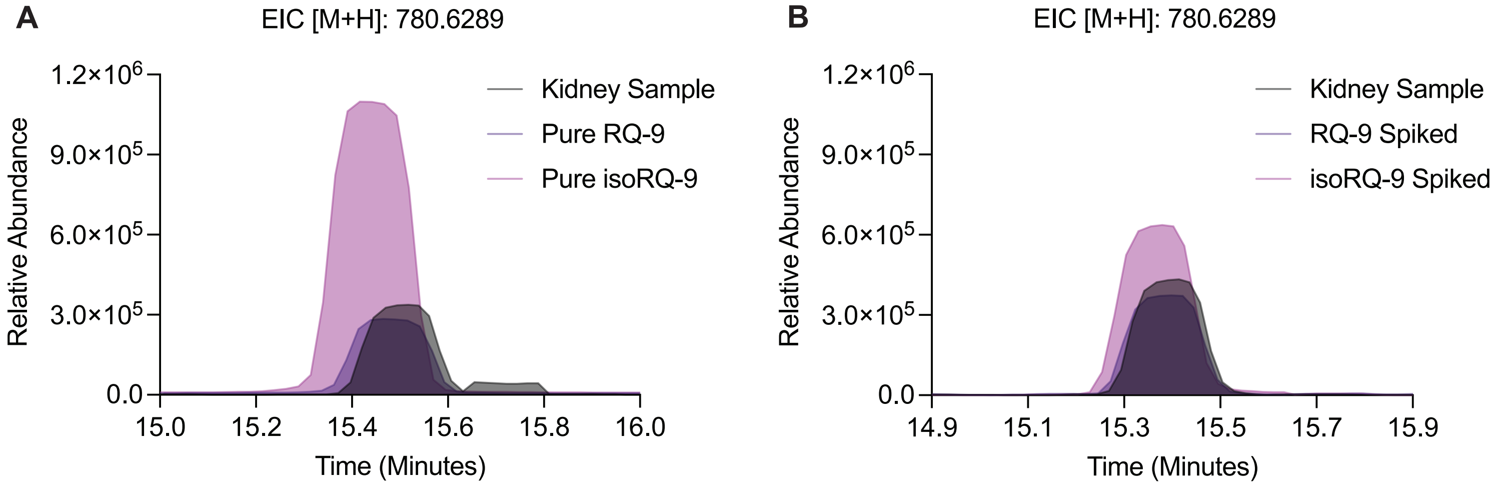
A
EIC [M+H]: 780.6289
1.2×10^6
9.0×10^5
6.0×10^5
3.0×10^5
0.0
Relative Abundance
15.0
15.2
15.4
15.6
15.8
16.0
Time (Minutes)
Kidney Sample
Pure RQ-9
Pure isoRQ-9
B
EIC [M+H]: 780.6289
1.2×10^6
9.0×10^5
6.0×10^5
3.0×10^5
0.0
Relative Abundance
14.9
15.1
15.3
15.5
15.7
15.9
Time (Minutes)
Kidney Sample
RQ-9 Spiked
isoRQ-9 Spiked
