# Supplementary material for: A method to synthesize analytical rhodoquinone standards for quantitative analysis in tissue specimen
Source: bioRxiv. 2026 May 7:2026.05.04.722805. Preprint. [Version 1] doi: 10.64898/2026.05.04.722805 (PMC13174367; doi:10.64898/2026.05.04.722805)
Supplement: Supplementary file 1 [file NIHPP2026.05.04.722805V1-supplement-1.pdf]

Figure S1

^{1}H NMR ($CDCl_3$, 500 MHz)
Compound 1
isoRhodoquinone-10

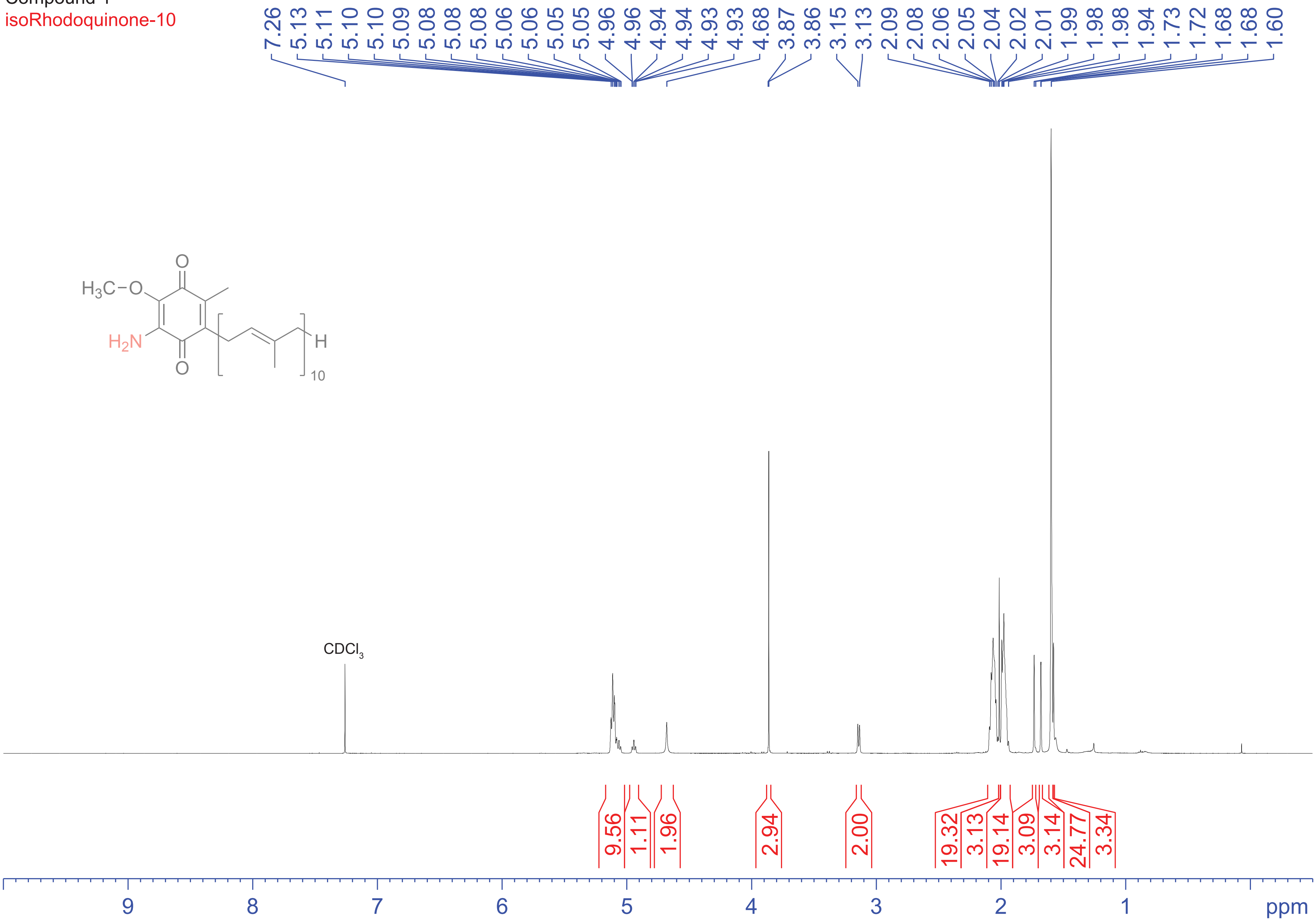

Figure S2

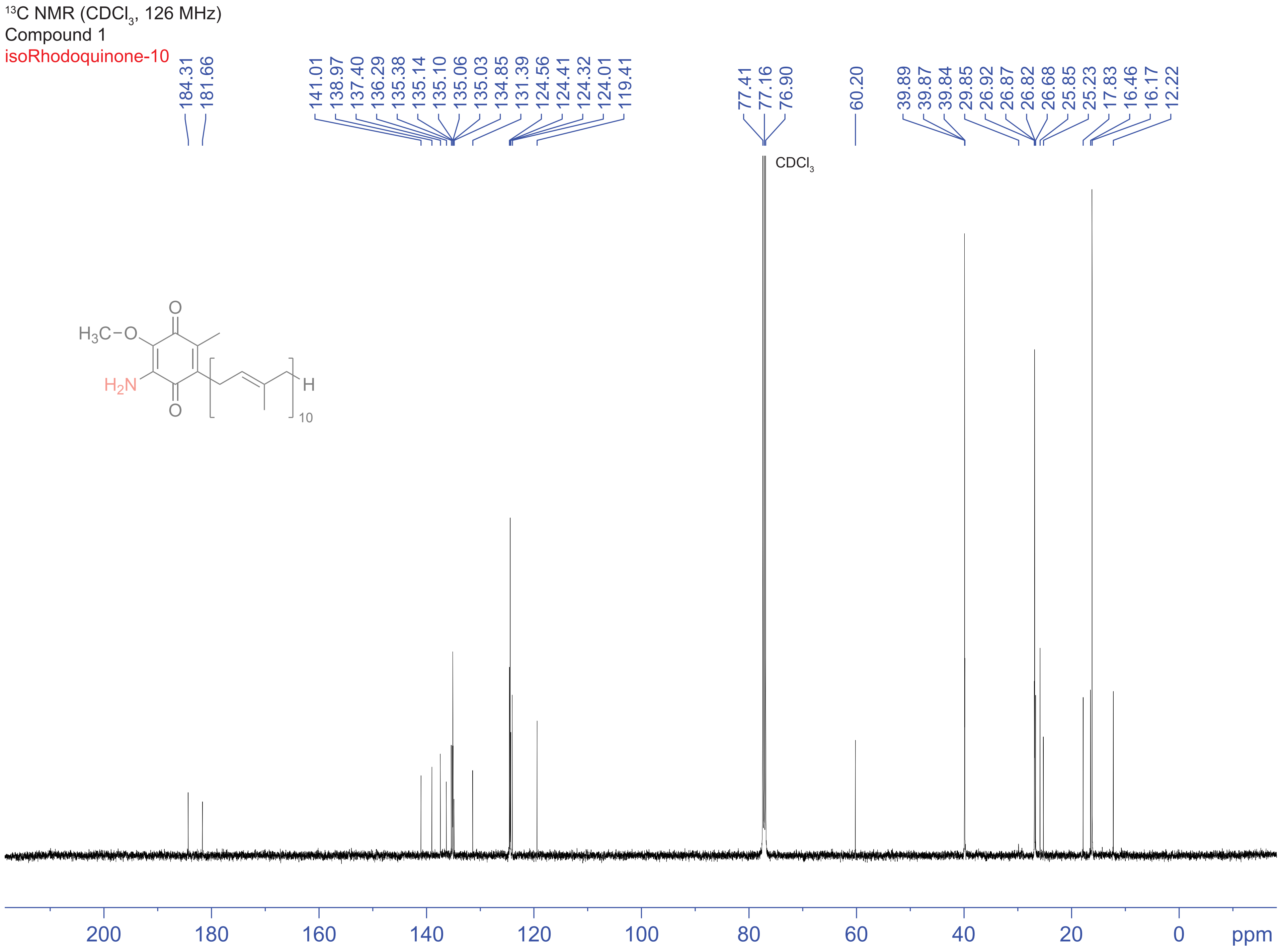
13C NMR (CDCl3, 126 MHz)
Compound 1
isoRhodoquinone-10
184.31
181.66
141.01
138.97
137.40
136.29
135.38
135.14
135.10
135.06
135.03
134.85
131.39
124.56
124.41
124.32
124.01
119.41
77.41
77.16
76.90
60.20
39.89
39.87
39.84
29.85
26.92
26.87
26.82
26.68
25.85
25.23
17.83
16.46
16.17
12.22
CDCl3
H3C−O
H2N
O
H
10
200
180
160
140
120
100
80
60
40
20
0
ppm

Figure S3

DEPT NMR ($CDCl_3$, 126 MHz)
Compound 1
isoRhodoquinone-10

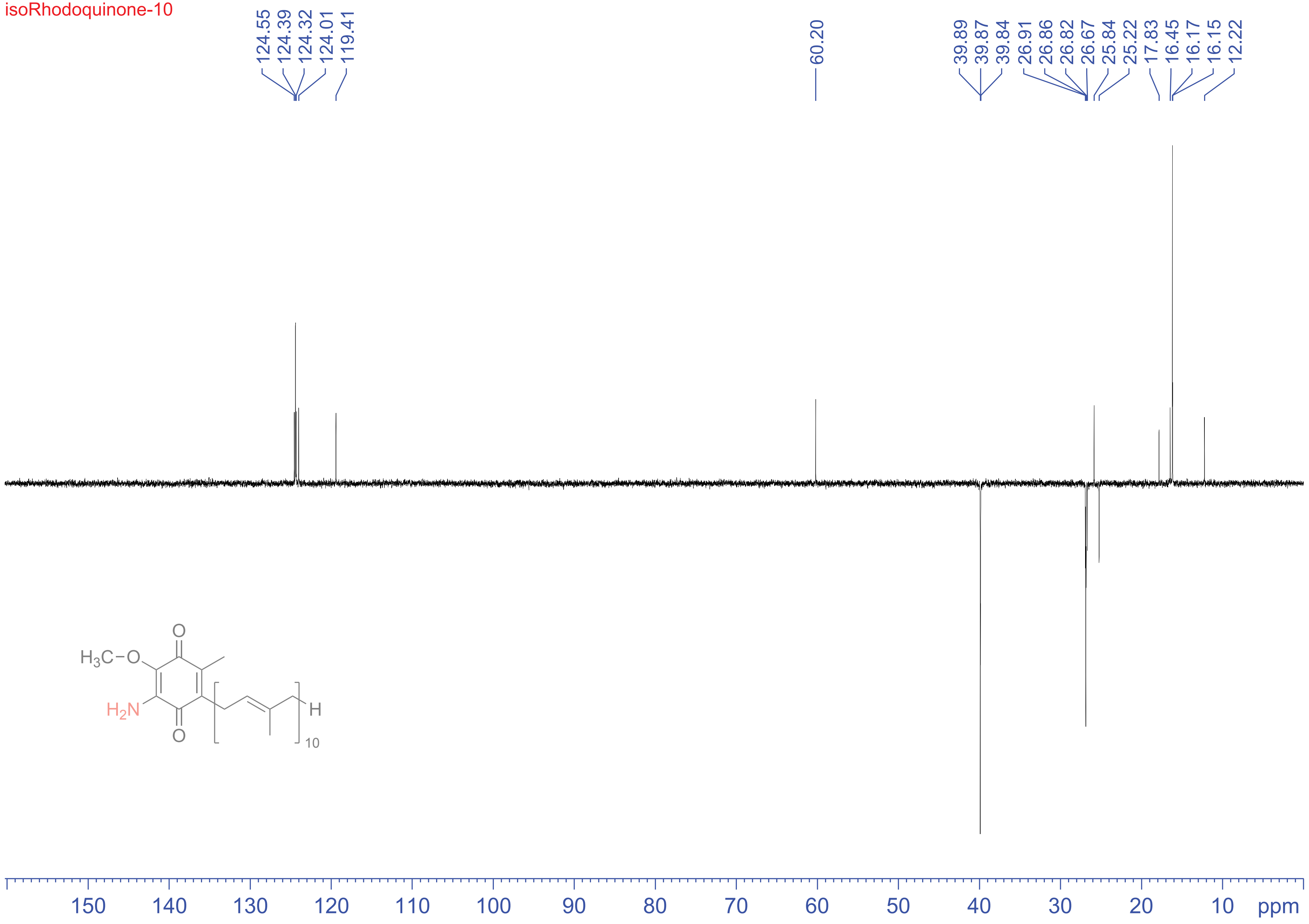

Figure S4

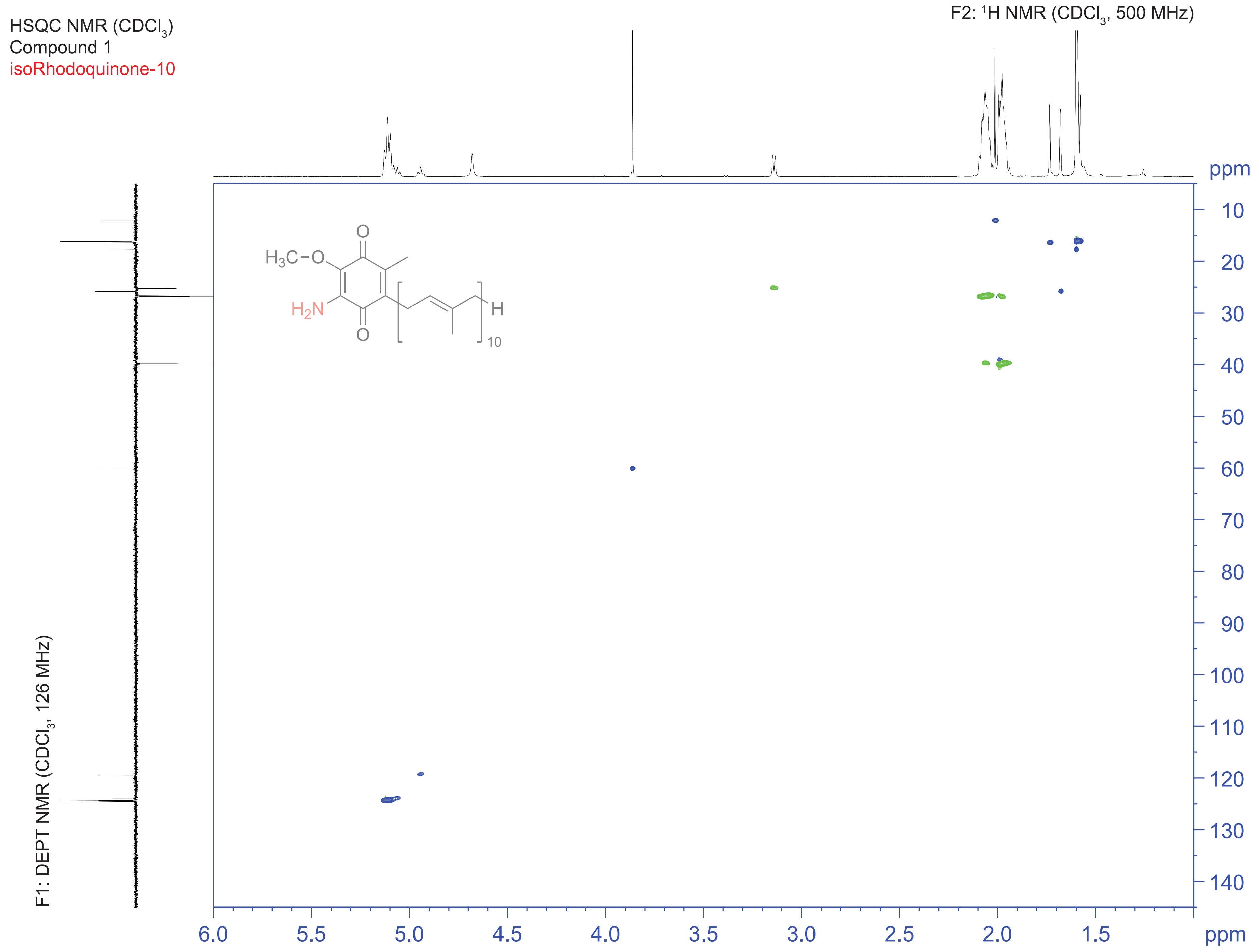
HSQC NMR (CDCl3)
Compound 1
isoRhodoquinone-10
F2: 1H NMR (CDCl3, 500 MHz)
F1: DEPT NMR (CDCl3, 126 MHz)
H3C−O
H2N
O
H
10
ppm
10
20
30
40
50
60
70
80
90
100
110
120
130
140
6.0
5.5
5.0
4.5
4.0
3.5
3.0
2.5
2.0
1.5

Figure S5

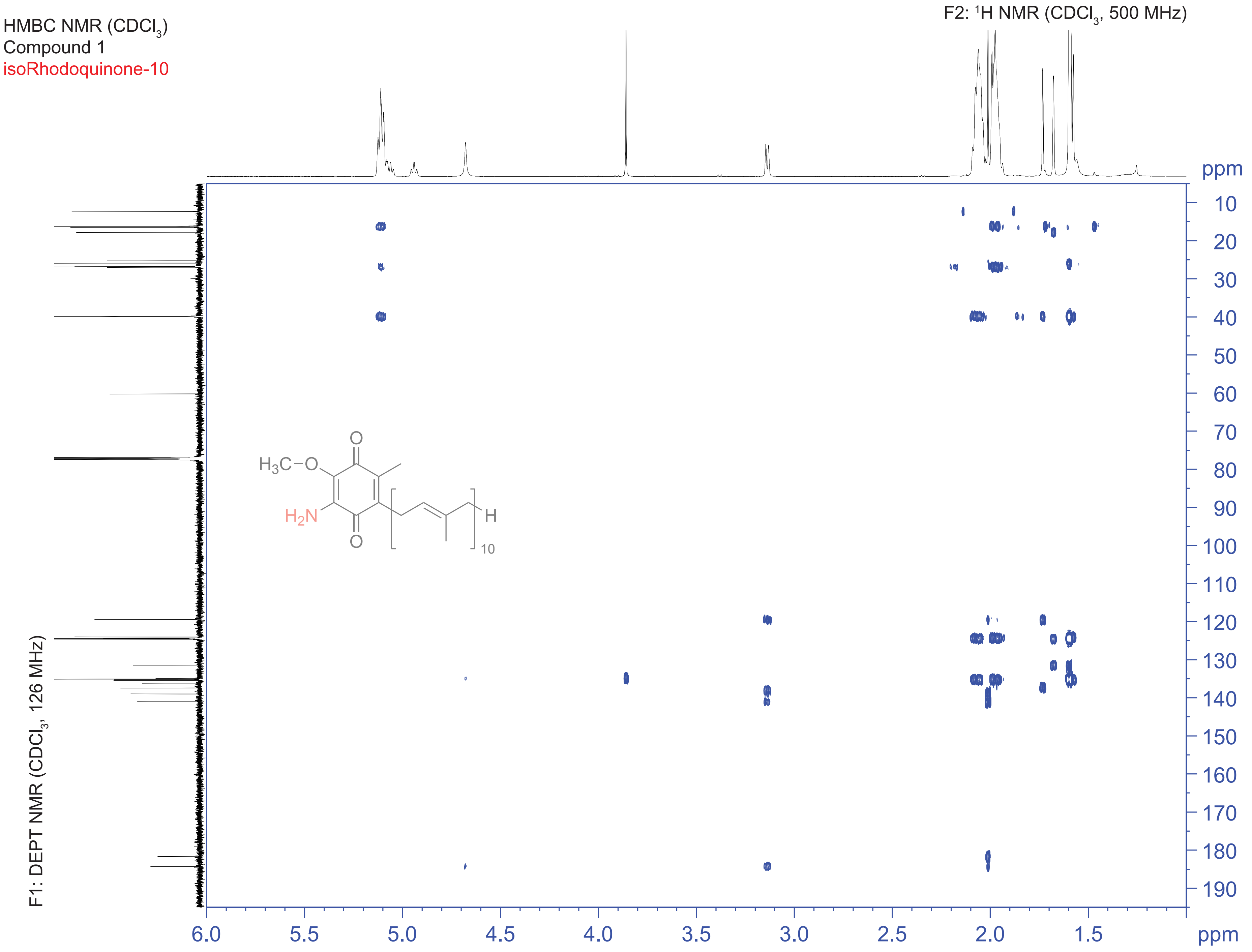

HMBC NMR (CDCl3)
Compound 1
isoRhodoquinone-10
F2: 1H NMR (CDCl3, 500 MHz)
F1: DEPT NMR (CDCl3, 126 MHz)
ppm
10
20
30
40
50
60
70
80
90
100
110
120
130
140
150
160
170
180
190
6.0
5.5
5.0
4.5
4.0
3.5
3.0
2.5
2.0
1.5
ppm
H3C−O
O
O
H2N
H
10

Figure S6

^{1}H NMR ($CDCl_3$, 500 MHz)
Compound 2
Rhodoquinone-10

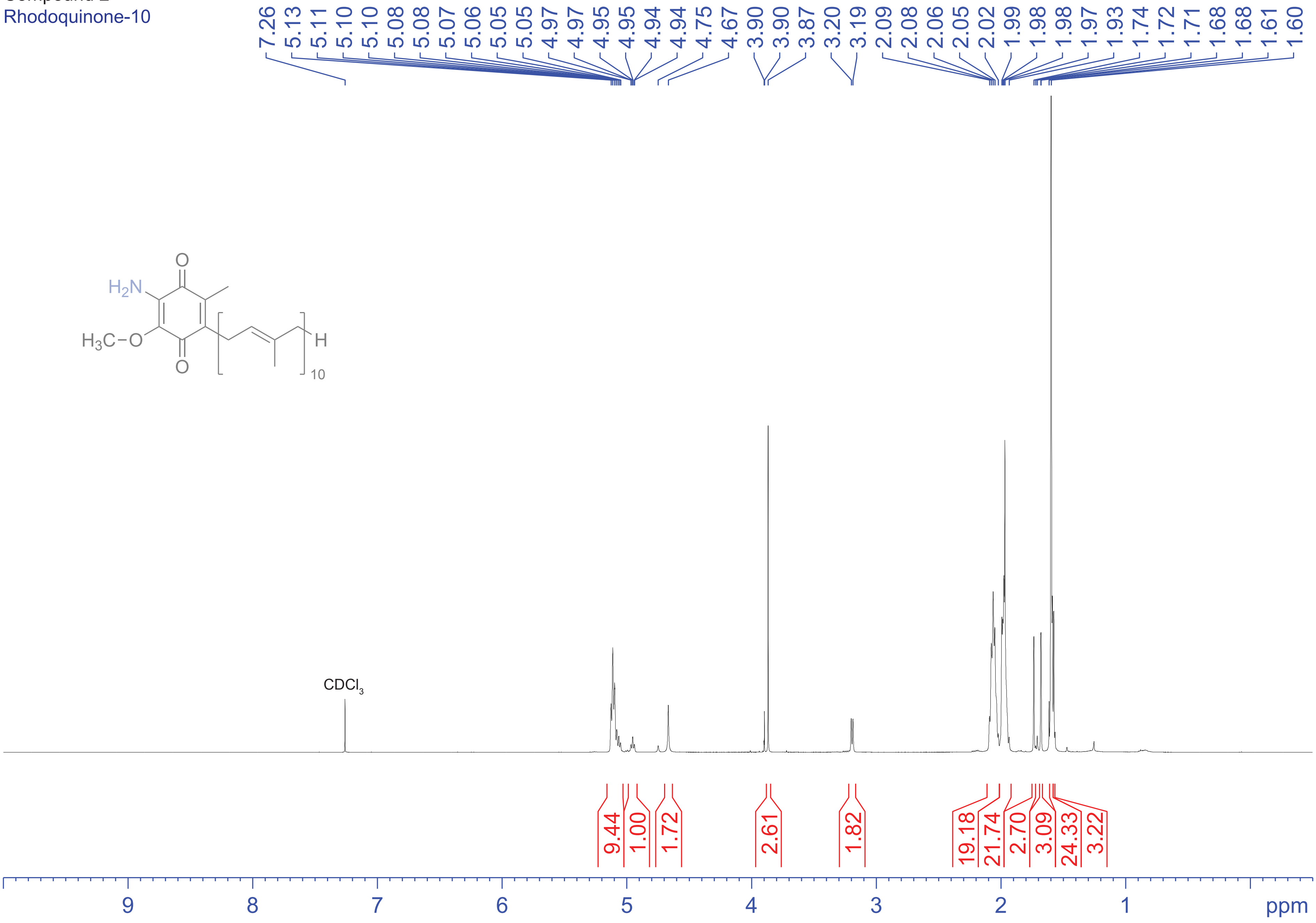

Figure S7

^{13}C NMR ($CDCl_3$, 126 MHz)
Compound 2
Rhodoquinone-10

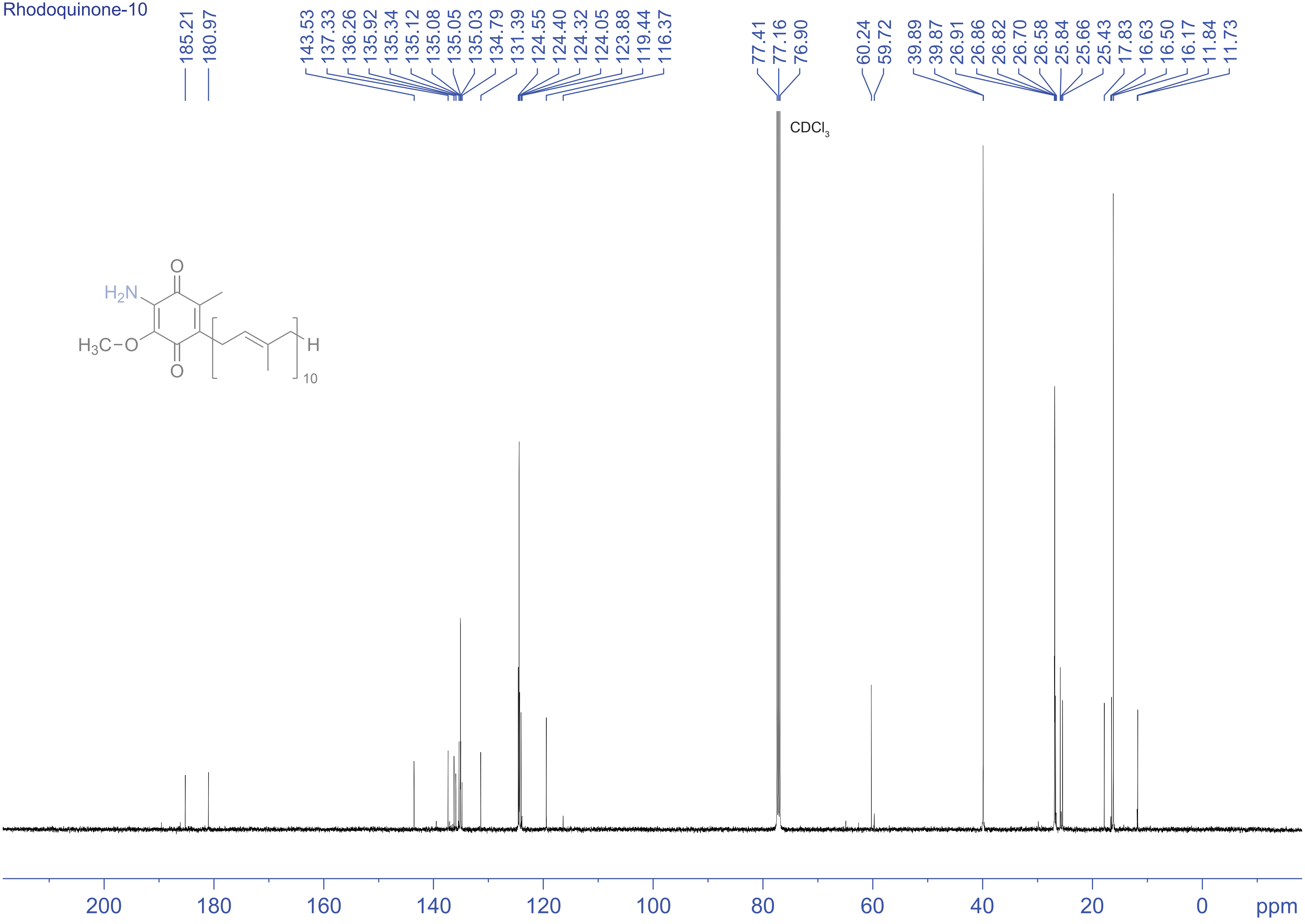

Figure S8

DEPT NMR ($CDCl_3$, 126 MHz)
Compound 2
Rhodoquinone-10

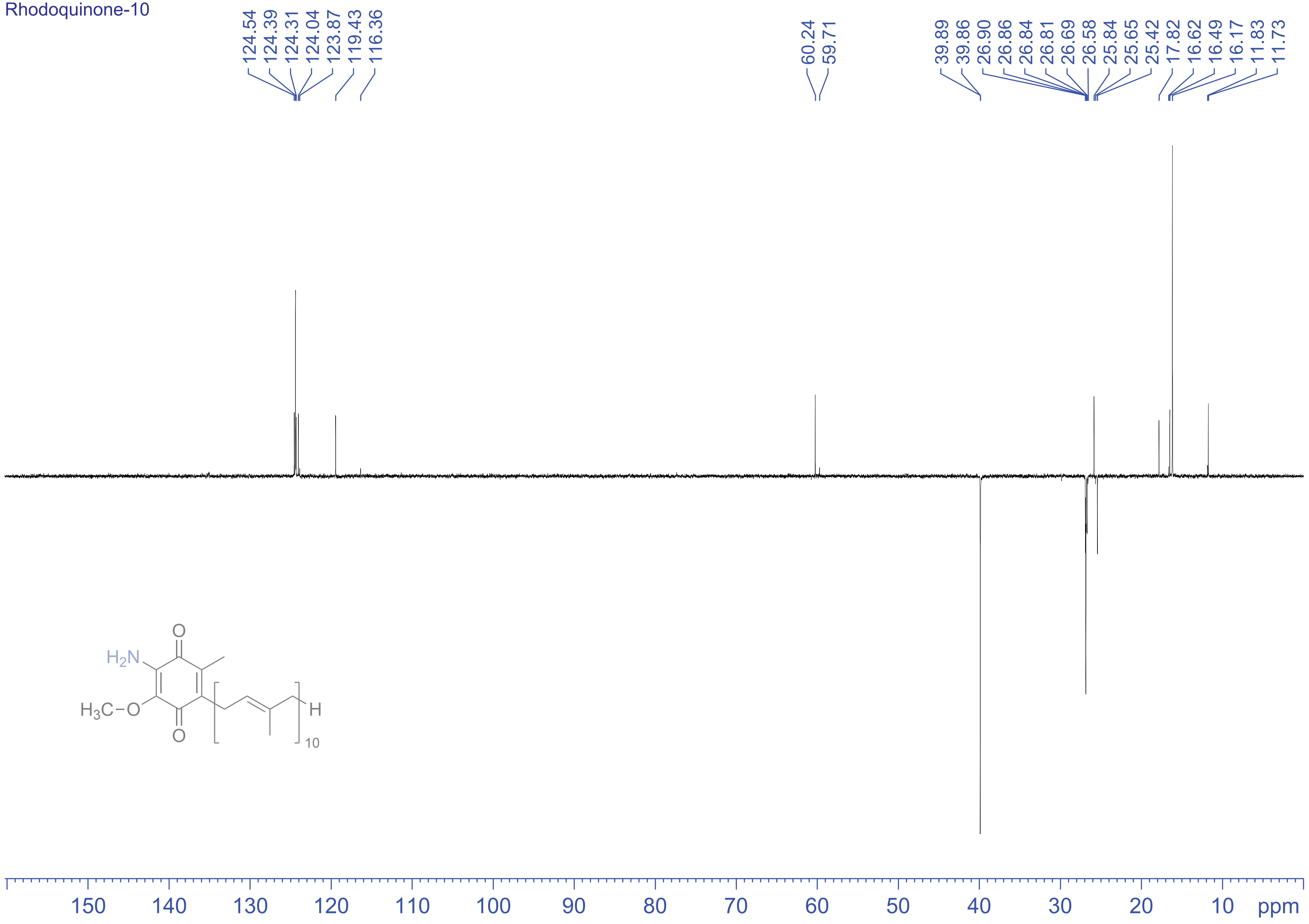

Figure S9

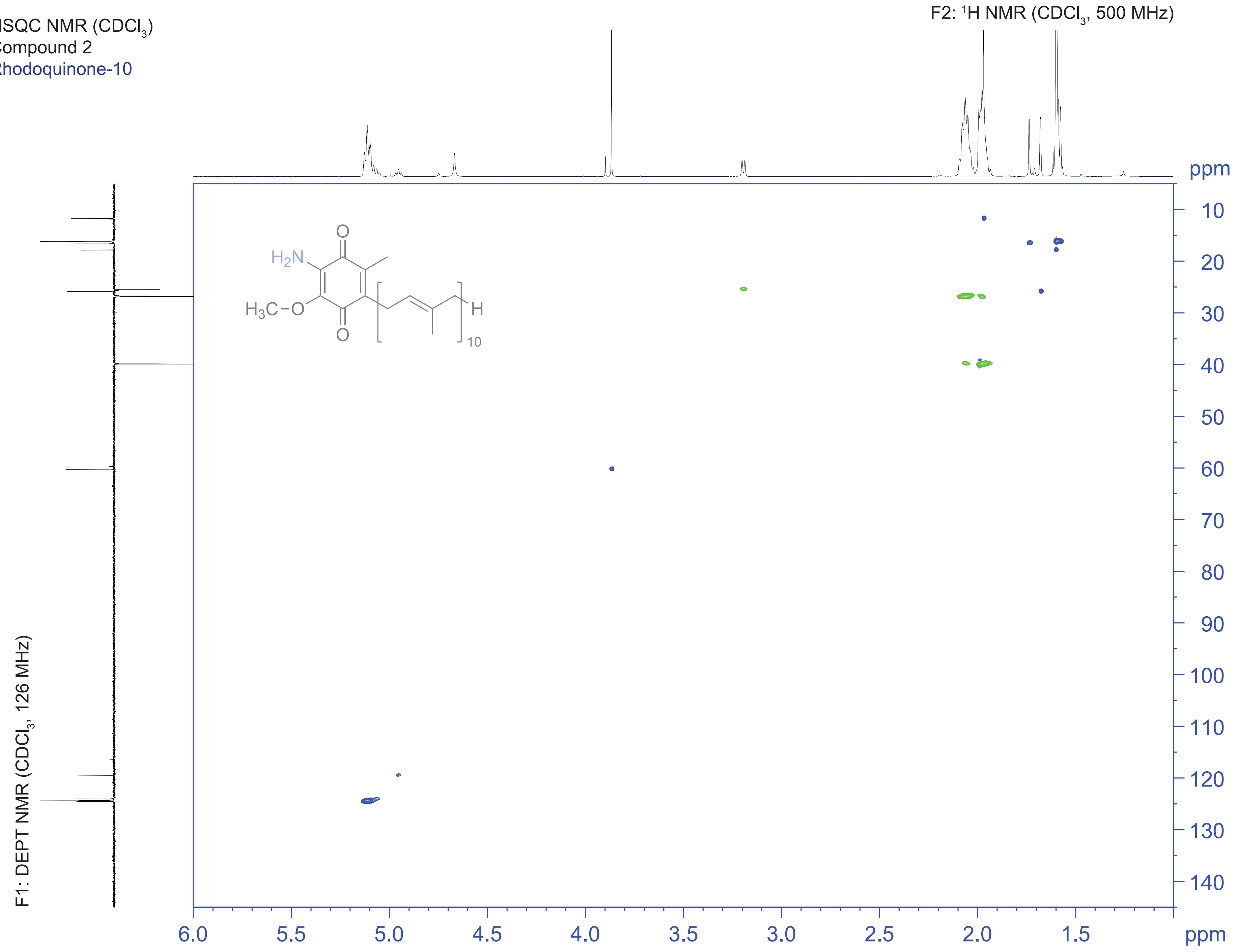
HSQC NMR ($CDCl_3$)
Compound 2
Rhodoquinone-10
F2: ^{1}H NMR ($CDCl_3$, 500 MHz)
F1: DEPT NMR ($CDCl_3$, 126 MHz)
H_2N
H_3C-O
O
O
H
10
ppm
10
20
30
40
50
60
70
80
90
100
110
120
130
140
6.0
5.5
5.0
4.5
4.0
3.5
3.0
2.5
2.0
1.5
ppm

Figure S10

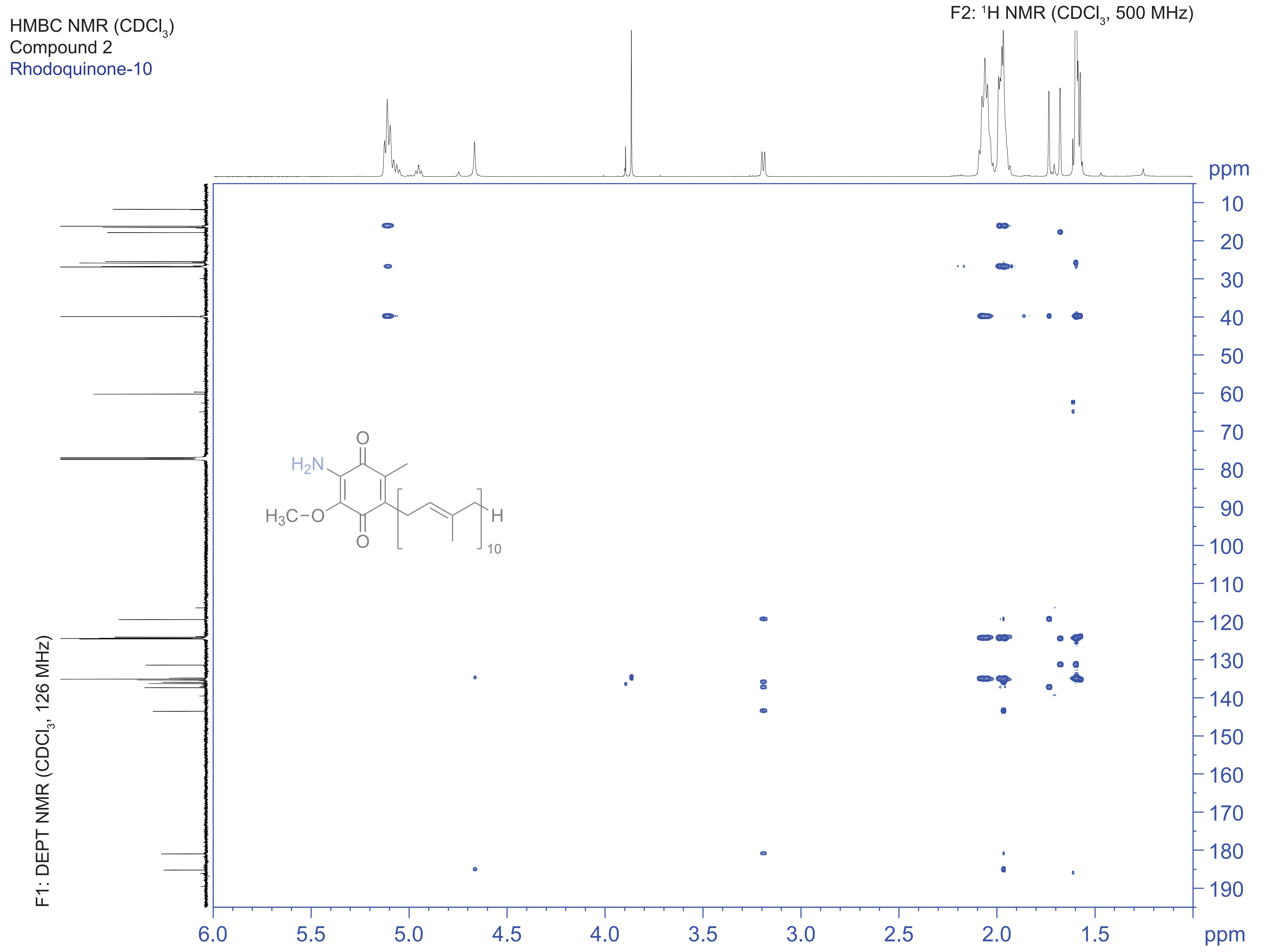
HMBC NMR ($CDCl_3$)
Compound 2
Rhodoquinone-10
F2: ^1H NMR ($CDCl_3$, 500 MHz)
F1: DEPT NMR ($CDCl_3$, 126 MHz)
ppm
10
20
30
40
50
60
70
80
90
100
110
120
130
140
150
160
170
180
190
6.0
5.5
5.0
4.5
4.0
3.5
3.0
2.5
2.0
1.5
ppm
O
H_2N
H_3C-O
O
H
10

bioRxiv preprint doi: https://doi.org/10.64898/2026.05.04.722805; this version posted May 7, 2026. The copyright holder for this preprint (which was not certified by peer review) is the author/funder, who has granted bioRxiv a license to display the preprint in perpetuity. It is made available under aCC-BY 4.0 International license.

Figure S1. Proton (^{1}H) NMR (500 MHz, $CDCl_3$) spectra of isorhodoquinone-10.
Figure S2. Carbon 13 (^{13}C) NMR (126 MHz, $CDCl_3$) spectra of isorhodoquinone-10.
Figure S3. Distortionless Enhancement by Polarization Transfer (DEPT) NMR (126 MHz, $CDCl_3$) spectra of isorhodoquinone-10.
Figure S4. Heteronuclear Single Quantum Coherence (HSQC) NMR analysis of isorhodoquinone-10.
Figure S5. Heteronuclear Multiple Bond Correlation (HMBC) NMR analysis of isorhodoquinone-10.
Figure S6. Proton (^{1}H) NMR (500 MHz, $CDCl_3$) spectra of rhodoquinone-10.
Figure S7. Carbon 13 (^{13}C) NMR (126 MHz, $CDCl_3$) spectra of rhodoquinone-10.
Figure S8. Distortionless Enhancement by Polarization Transfer (DEPT) NMR (126 MHz, $CDCl_3$) spectra of rhodoquinone-10.
Figure S9. Heteronuclear Single Quantum Coherence (HSQC) NMR analysis of rhodoquinone-10.
Figure S10. Heteronuclear Multiple Bond Correlation (HMBC) NMR analysis of rhodoquinone-10
